# Pelvis and femur shape prediction using principal component analysis for body model on seat comfort assessment. Impact on the prediction of the used palpable anatomical landmarks as predictors

**DOI:** 10.1371/journal.pone.0221201

**Published:** 2019-08-27

**Authors:** Léo Savonnet, Sonia Duprey, Serge Van Sint Jan, Xuguang Wang

**Affiliations:** 1 Univ Lyon, Université Claude Bernard Lyon 1, Lyon, France; 2 Laboratory of Anatomy, Biomechanics and Organogenesis (LABO) of Université Libre de Bruxelles (ULB), Brussels, Belgium; Virginia Tech, UNITED STATES

## Abstract

A personalized pelvis and femur shape is required to build a finite element buttock thigh model when experimentally investigating seating discomfort. The present study estimates the shape of pelvis and femur using a principal component analysis (PCA) based method with a limited number of palpable anatomical landmarks (ALs) as predictors. A leave-one-out experiment was designed using 38 pelvises and femurs from a same sample of adult specimens. As expected, prediction errors decrease with the number of ALs. Using the maximum number of easily palpable ALs (13 for pelvis and 4 for femur), average errors were 5.4 and 4.8 mm respectively for pelvis and femur. Better prediction was obtained when the shapes of pelvis and femur were predicted separately without merging the data of both bones. Results also show that the PCA based method is a good alternative to predict hip and lumbosacral joint centers with an average error of 5.0 and 9.2 mm respectively.

## Introduction

Estimating bone shapes and locating joint centers from external palpable anatomical landmarks (ALs) are required for biomechanical analysis in many applications [1],[2]. This is particular true when a finite element human body model is used to estimate the internal strain in soft tissue for assessing seating discomfort (see a recent review [3]). However reconstructing 3D bone geometry usually requires imaging techniques (MRI, CT scan, etc…) which is expensive, time-consuming and not always available. To estimate the bone shape, several methods have been proposed. The first and simplest one is to scale a reference model as proposed in [4] only using two scaling factors, one related to segment length and the other to body mass. An alternative is the kriging [5], which deforms a surface by controlling a set of points to match their target positions as performed by [6]. However, a high number of control points are required and this method is not applicable when only a small number of palpable landmarks are available. In recent years, principal component analysis (PCA) based methods were used to analyze the shape variation of external skin (e.g.[7], [8]), and also internal organs (e.g. [9], [10]) as well as the relationship between external body shape and internal joint centers’ position [11]. PCA-based statistical shape prediction methods have also been proposed especially in the field of computer-assisted orthopedic surgery, where 3D patient-specific anatomy has to be recovered from incomplete information. The information could be anthropometric dimensions, clinically and surgically relevant morphometric parameters [12], sparse surface points [13] or 2D fluoroscopic images [14]. In comparison with orthopedic surgery applications, no medical images or bone surface points are generally available in other applications such as seating comfort studies. Only a very limited number of palpable ALs can be measured. How good is the reconstructed bone surface from such a limited number of ALs? This question was partially investigated by [15] in case of pelvic shape reconstruction. In this study, different set of landmarks were picked from the bone surface to be predicted and used as predictors. However, the unknown bone surface was already aligned with the rest of training data using all surface points. Larger errors are certainly expected when only a limited number of ALs is available because of the uncertainty in landmark palpation and misalignment using a limited number of ALs.

The objective of the present study is therefore to evaluate a PCA based method to estimate the shape of pelvis and femur from a limited number of palpable ALs, as the present work is mainly motived by developing personalized finite element buttock-thigh model for assessing seating comfort.

## Material and methods

**Fig 1:**
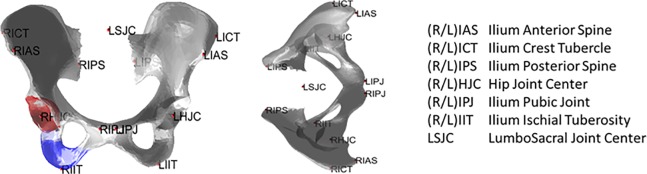
Definition of the palpable anatomical landmarks for the pelvis (R = Right, L = Left).

**Fig 2:**
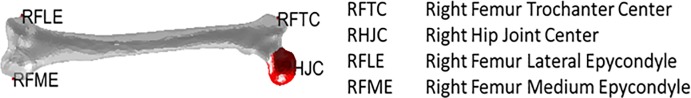
Definition of the palpable anatomical landmarks for the femur.

54 CT-scans of pelvis and femur obtained from the Body Donation program by the ULB (Université Libre de Bruxelles) [16] were used in the present study. The scans were performed in a supine position with a Siemens OMATUM scanner. The pelvis and femur images were segmented using a commercially available software program (Amira) to obtain 3D surfaces of the bones of interest. 13 landmarks on the pelvis (Fig 1) and 4 landmarks on the femur (Fig 2) were virtually palpated on the reconstructed surfaces [17] except the hip (RHJC, LHJC) and lumbosacral (LSJC) joint centers which were estimated indirectly [16].

The raw data was first cleaned by filling the holes and deleting the superimposed mesh parts. Thirty-eight specimens were kept after elimination of the scans with too many holes or crossing meshes. A generic template was created manually from one of the available specimens and then it was deformed to match the scanned surface of each subject using mHBM software (Markerless Homologous Body Modeling Software, National Institute of Advanced Industrial Science and Technology, Digital Human Research Center). At the end we obtained 38 pairs of pelvis and related femoral bones with the same number of vertices (5183 vertices) aligned to their respective average shape using the palpable landmarks.

A PCA model [18] was built on the coordinates of the vertices and palpated ALs of the 38 pairs of pelvis and femur. A bone shape can be described as the mean shape plus a weighted linear combination of the eigenvectors of the covariance matrix. To predict the bone shape from a set of palpable landmarks, we used the algorithm proposed by [13], which minimizes the Euclidean distance between the predicted and the target landmarks as well as a second term which is the Mahalanobis distance of the predicted shape from the mean shape. The second term ensures that the predicted shapes do not deviate from the mean. A weighting coefficient ρ should be defined to find a good compromise between these two terms. In [13], they suggest ρ = 0.5 when the number of target points is smaller than 6. For the pelvis, 13 ALs were defined if the two hip joint centers and lumbosacral center are included. Due to the symmetry, only 7 ALs can be considered independent. For the femur, 4 ALs were defined including hip joint center. In the present work, ρ = 0.5 was used for both pelvis and femur. Thanks to the second term, the scores corresponding to the principal components (PCs) explaining secondary shape variations were determined to maintain the predicted shape closer to the mean of the training data. Different numbers of PCs were tested. The number of PCs explaining 95% of variance was selected for both the pelvis (20 PCs) and femur (10 PCs), as a higher number of PCs did not improve the predictions.

Other factors affect the prediction of bone shape. The number of ALs used as predictors is certain one of main factors. As pelvis and femur data were from the same specimen, PCA models with combined pelvis and femur data were also developed and compared with their respective separated model. A numerical experimental design was built to study the effect of each of these parameters. A leave-one-out procedure was applied for each combination of these factors. The PCA model was first built from n-1 specimens, and then the bone shape of the n^th^ extra specimen was predicted only using the digitally palpated landmarks of this specimen. For each subject, the distance between the original and predicted surfaces was computed. This LOO procedure was repeated 38 times and the average distance over the 38 repetitions was computed. Three surfaces of interest for the pelvis were analyzed (colored on Fig 1): whole surface, ischial (in blue) and acetabular (in red) areas. Two surfaces were analyzed for the femur (Fig 2): whole surface and femoral head (in red). The distance between the palpated and predicted landmarks was also computed.

## Results

**Fig 3:**
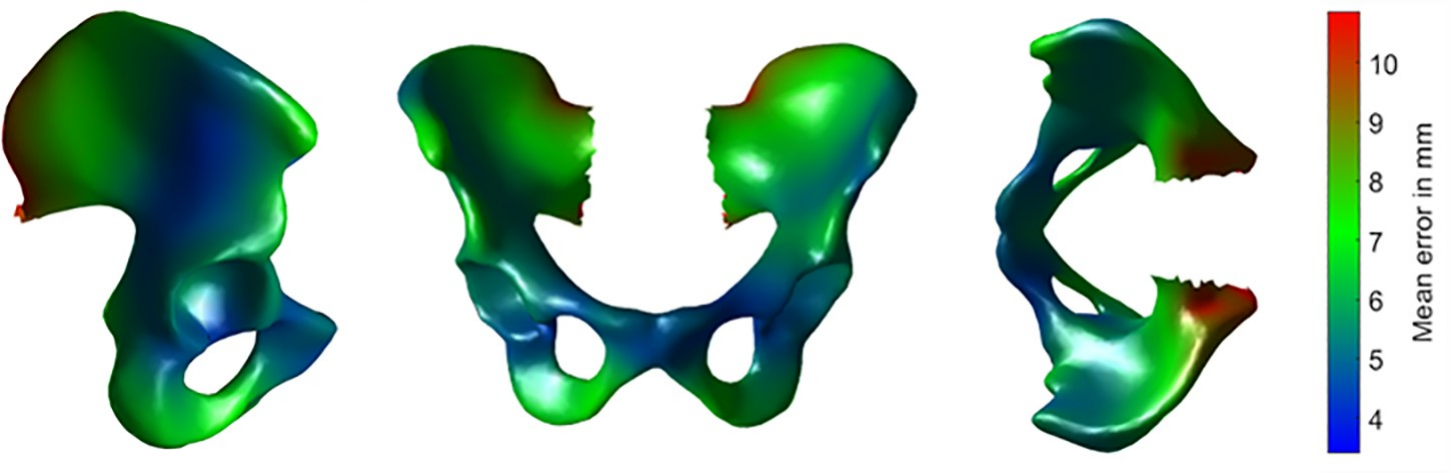
Mean error of the pelvis surface over the 38 specimens from the leave-one-out studies using the 13 landmarks.

**Fig 4:**
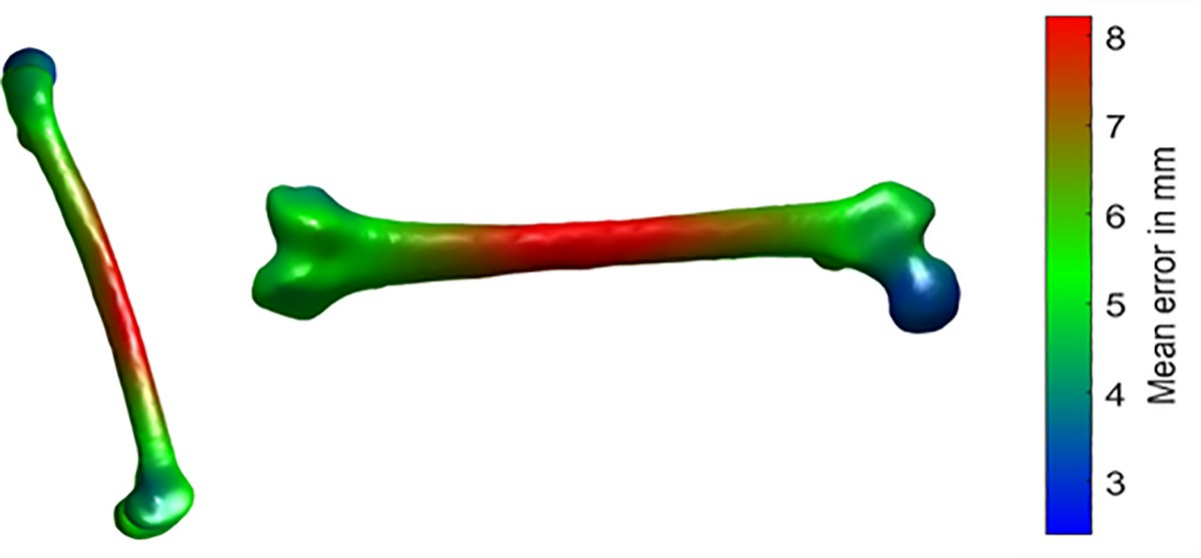
Mean error of the femur surface over the 38 specimens from the leave-one-out studies using the 4 landmarks.

As expected, the errors decreased with the number of ALs for the pelvis and the femur for all surfaces of interest (Tables 1 and 2). The smallest error for the whole surface was 5.4 mm and 4.8 mm on average respectively on the pelvis and femur when all available ALs were used without merging pelvis and femur data (Figs 3 and 4). For the pelvis, the acetabular area had the smallest error of 3.4 mm while the largest error of 10.8 mm was observed at the bottom of the ischial crest. The error on the femur was small at the extremities and large at the middle. The minimum of 2.4 mm was observed on the femoral head while the maximum was 8.2 mm at the middle area.

**Table 1:** Distances (mm) between original and predicted surfaces and ALs from the leave-one-out experiment for the pelvis. N°8 was obtained using combined data of pelvis and femur, whereas only the pelvis data were used for building PCA models for the others.

		Whole		Ischium		Acetabulum		All ALs	RHJC	LSJC
N°	Landmarks	Mean	Maximum	Mean	Maximum	Mean	Maximum	Mean±SD	Mean±SD	Mean±SD
1	RIAS,LIAS,RICT,LICT	7.1	18.3	8.4	11.9	5.5	8.4	8.3 ± 2.9	5.5 ± 2.5	9.4 ± 3.4
2	N°1 + RIPS,LIPS	6.9	18.4	8.7	12.1	5.7	8.7	7.6 ± 3.1	5.5 ± 2.3	9.5 ± 3.8
3	N°2 + RIPJ,LIPJ	6.3	17.1	7.9	11.1	5.6	8.6	6.2 ± 3.5	5.5 ± 2.3	9.2 ± 4.2
4	N°3+ RHJC,LHJC	6.0	17.0	7.3	10.5	4.7	7.5	5.7 ± 1.5	-	9.3 ± 4.2
5	N°3 + RIIT,LIIT	5.7	16.9	5.8	8.5	5.2	7.9	4.4 ± 1.6	5.0 ± 2.2	9.2 ± 4.6
6	N°5 + RHJC,LHJC	5.5	16.9	5.9	8.5	4.4	7.0	4.3 ± 1.5	-	9.0 ± 4.4
7	N°6 + LSJC	5.4	16.6	5.7	8.5	4.5	7.1	4.1 ± 0.4	-	-
8	Combined + all ALs	5.5	16.0	5.8	8.5	4.7	7.2	4.8 ± 1.4	-	-

**Table 2:** Distances (mm) between original and predicted surfaces and ALs from the leave-one-out experiment for the femur. N°3 was obtained using combined data of pelvis and femur, whereas only the femur data were used for building PCA models for N°1 and N°2.

		Whole		Femoral head		All ALs	RHJC
N°	Landmarks	Mean	Maximum	Mean	Maximum		
1	RFTC,RFLE,RFME	5.8	13.1	7.0	9.9	4.3 ± 1.6	6.8 ± 3.8
2	N°1 + RHJC	4.8	11.0	2.9	5.3	1.5 ± 0.2	-
3	Combined + all ALs	5.8	13.5	5.1	7.8	3.6 ± 1.5	-

Predicted hip (HJC) and lumbosacral joint (LSCJ) centers were also compared with the measured ones when they were not used as predictors. Prediction errors seemed not much affected by the number of ALs for both joint centers, though the lowest error was obtained for both. The smallest error was 5 and 9 mm respectively for RHJC and LSJC when all available ALs were used (N°5 and N°6 of Table 1).

## Discussion and conclusion

A PCA-based method was developed to predict the pelvis and femur shape from palpable ALs. As expected, the prediction error decreased with the number of ALs. This can be also explained by a better alignment since the error of alignment is reduced when more landmarks are added. Another trivial observation is that the prediction error per tested area was reduced when information (ALs) was added from the respective area. The error is much reduced when the ALs at the pubis symphysis (RIPJ and LIPJ), the ischia (ITT) or hip joint centers (HJC) are added, showing the importance of having these ALs. However, prediction error is not much affected by the landmarks R/LIPS or LSJC (Table 1). This is probably because the CT images in the sacral arear were very noisy leading to the difficult palpation of those specific landmarks. For the femur, the error was maximal at the center, which is the most distant area from the landmarks. The use of the hip joint center (HJC) much reduces the prediction error of the femoral head surface.

When comparing our results with those from [15], a higher error was found. They observed an error of 3.65 mm for the pelvis shape prediction with four landmarks. One reason could be that they aligned the pelvis with the whole surface whereas we only used the selected landmarks to align the surface to be predicted. The variance of the data sample they used was much lower with an average error of 4.86 mm between specimens and a mean pelvic shape instead of 7.5 mm in our study.

The PCA based shape prediction method can also predict the landmarks and joint centers. As for the surface, the smallest prediction error was obtained when the maximal number of the ALs was used as predictors. An error of 5.0 mm and 9.2 mm on average was obtained respectively for the hip (HJC) and lumbosacral (LSJC) joint centers when all landmarks excluding hip and lumbosacral joint centers. In our previous study [16], the same datasets were used to propose the regression equations of the distances between these two joint centers and ALs. The best prediction was obtained when using RIAS, LIAS and RIPS and IPJ with an average error of 7.0 and 8.6 mm respectively for HJC and LSJC. Compared to the results from [16], a better prediction was obtained for HJC while a slightly larger error was found for LSJC. This is probably due to the difficulty in locating this point because of the high noise in this area.

Pelvis and femur datasets were also merged together to build the PCA model. However, slightly higher prediction errors were obtained for both pelvis and femur using merged data. Higher error is induced by the use of the ALs of the adjacent bone. For instance, for the femur head, the average error is 5.1 mm when combining the data of pelvis and femur; whereas it is 2.9 mm when datasets are used separately. Consequently, it is recommended to predict bone surface only using the PCA model from separate training datasets.

In practice, it is recommended that a maximum number of palpable ALs are used to reduce bone shape prediction error. For the pelvis, in addition to the easily palpable landmarks at the iliac crest (RIAS, LIAS, RICT, LICT), the pubic symphysis (RIPJ, LIPJ) should be palpated. The ischial tuberosity could also be detected with help of a pressure map by locating the peak pressure [19]. Of course, hip joint centers cannot be palpated directly. They can be indirectly estimated either using other ALs as suggested in [16] or functionally by motion analysis [20]. For the femur, three ALs are easily palpable. As already stated in [16] and in [1],[2], accurately locating bone landmarks is always challenging especially for overweight or obese subjects. It is highly recommended that experimenters follow the strict definition of palpable ALs proposed in the literature [17].

The presented method can be used as a first step to develop a personalized buttock thigh finite element model with help of a limited number of palpated bony landmarks. The external skin shape can be easily scanned using a 3D scanner. The anatomical landmarks can be palpated manually and merged with the surface scan using a local technical coordinate system. Personalized pelvis and femur bone surfaces can then be generated using the method proposed in the present paper with the palpated landmarks as inputs. As CT-scans were done in a supine position, the pelvis and femur have to be repositioned in a desired seated position with help of the palpated landmarks assuming the hip as a spherical joint. In case of a simplified FE model with soft tissues being represented as a homogenous material, personalized skin and bone surfaces can then be directly meshed to create corresponding FE model. If a model with more detailed soft tissues (muscles, fat, ligaments…) is needed, a generic template containing these geometries could be morphed to match pelvis and skin geometry by kriging as proposed in the PIPER project ([21], www.piper-project.org).

In summary, the present study proposes a PCA based method to predict 3D pelvis and femur bone shapes only from a limited number of palpable anatomical landmarks and shows that the proposed method can predict pelvis and femur shapes with an error lower than 6 mm on average only using the palpable ALs including indirectly estimated hip joint centers. Of course, higher errors are expected when the ALs are physically palpated on living persons. Depending on operator and subject, uncertainty in locating ALs should be much higher than digital palpation used in the present study especially for subjects with high body mass index. The accuracy of reconstructed bone shape from physically palpated anatomical landmarks has not been investigated in this study, but should be examined in the future.

## Supporting information

S1 File Res_TOTAL_surface_prediction. Distances (mm) between original and predicted surfaces from the leave-one-out experiment for the pelvis.
(XLSX)

S2 File Res_TOTAL_landmarks_prediction_revised. Distances (mm) between original and predicted ALs from the leave-one-out experiment for the pelvis.
(XLSX)
